# Fishy Aroma of Social Status: Urinary Chemo-Signalling of Territoriality in Male Fathead Minnows (*Pimephales promelas*)

**DOI:** 10.1371/journal.pone.0046579

**Published:** 2012-11-07

**Authors:** Dalma Martinovic-Weigelt, Drew R. Ekman, Daniel L. Villeneuve, Channing M. James, Quincy Teng, Timothy W. Collette, Gerald T. Ankley

**Affiliations:** 1 Department of Biology, University of St. Thomas, St. Paul, Minnesota, United States of America; 2 Ecosystems Research Division, U.S. EPA, Athens, Georgia, United States of America; 3 Mid-Continent Ecology Division, U.S. EPA, Duluth, Minnesota, United States of America; Centre of Marine Sciences & University of Algarve, Portugal

## Abstract

Chemical structures of several urinary reproductive pheromones in fish have been identified, and their role in the chemical communication of reproductive condition is well characterized. On the contrary, the role of chemical communication in signalling of social/territorial status in fish is poorly understood. Fathead minnows are an example of a fish species whose life history traits appear conducive to evolution of chemical communication systems that confer information about social/territorial status. Male reproduction in this species is dependent upon their ability to acquire and defend a high quality nesting territory, and to attract a female to the nest. We hypothesized that fathead minnow males use visual and urine-derived chemical cues to signal territorial status. To test this hypothesis, effects of territorial acquisition on male-specific secondary sex characteristics (SSCs) and urine volumes were first assessed. Second, frequencies of male urination in varying social contexts were examined. Finally, nuclear magnetic resonance-based metabolomics was used to identify urinary metabolites that were differentially excreted in the urine of territorial versus non-territorial males. The expression of SSCs, sperm, and urine volumes increased with territory acquisition, and either remained unchanged or decreased in non-territorial males. Frequency of male urination increased significantly in the presence of females (but not males), suggesting that females are the main target of the urinary signals. Territorial and non-territorial males had distinct urinary metabolomic profiles. An unforeseen finding was that one could discern future territorial status of males, based on their initial metabolomic profiles. Bile acids and volatile amines were identified as potential chemical signals of social status in the fathead minnow. The finding that trimethylamine (a fishy smelling volatile amine) may be a social cue is particularly interesting, because it is known to bind trace amine-associated receptors, indicating that these receptors may play role in chemical signalling of social status in fish.

## Introduction

Urine is commonly used by both invertebrates and vertebrates as a carrier of a variety of chemosensory signals [1], [2]. In some aquatic animals chemosensory cues in urine are released in a controlled, pulsed manner, often synchronizing performance of behaviors (e.g., reproduction, aggression) in their conspecifics [3], [4]. Female fish are a well-studied chemical communication model, and have been shown to actively use urinary pheromones to communicate reproductive status to their conspecifics [5]. Chemical structures of several urinary reproductive pheromones in fish have been identified, and their role in the chemical communication of reproductive readiness is well characterized [6]–[8]. Compared to reproductive condition, the role of chemical communication in signalling of social status in fish is less understood. Use of urine-based chemical stimuli for communication of social status, and as a tool for manipulation of an opponent's intrinsic neural state and behavior has been demonstrated in aquatic invertebrates [9], [10], suggesting that this strategy might also be used by aquatic vertebrates. Recent studies of the role of chemical communication in social interactions suggest that at least two species of fish, Nile tilapia (*Oreochromis mossambicus*) and an African cichlid (*Astatotilapia burtoni*) may use urine to advertise social status amongst conspecific males, and females [11]–[14]. Barata et al. [13] proposed that an aminosterol-like odorant in the urine of male Nile tilapia is used to communicate a male's social dominance to females, but the exact identities of the chemicals involved in communicating social status in fish remain poorly elucidated.

Fathead minnows (*Pimephales promelas*) (FHMs) exhibit life history traits which may be conducive to evolution of systems that use chemical communication to confer information about an individual's social status. To reproduce, males must attract a female to the nest, where she will deposit eggs that the male maintains and protects until hatching. Reproduction in males of this species is dependent upon their ability to acquire and defend a high quality nesting territory in the presence of conspecific male competitors; non-territorial males of this species are not known to exhibit alternative reproductive strategies such as ‘sneaking’ [15]. Social status affects the seasonal timing of reproduction and the size distribution of the young, with territorial, dominant males reproducing first, and with greater success [15]. Dominant *P. promelas* males use aggressive behavioral displays to visually and physically suppress subordinate males, thus preventing them from holding territories and spawning with females [16]. Given the importance of social status in reproduction, and energetic costs associated with agonistic encounters, nest maintenance, and female courtship, it seems reasonable to hypothesize that male FHMs might use chemical signals to communicate their social/territorial status to the females, and/or to modulate interactions with conspecific males.

There is some evidence for use of chemical communication in the FHMs; for example, females can distinguish sexually mature and immature males based on chemical cues [17]. Based on this, it is plausible that dominant (territorial, T) and subordinate (non-territorial, NT) males too, may have different urinary chemical profiles that can be discriminated by the females. It is well known that social status has profound effects on the physiology and tissue-specific metabolite profiles in fish. For example, glycolitic capacity of muscles (e.g., [18]), concentrations of circulating cortisol, triglycerides (e.g., [19], [20]), androgens (e.g., [21]) and a variety of neuromodulators (e.g., [22], [23]) are affected by social status. It is therefore likely that these types of variations in hormone and metabolite profiles could be reflected in chemical profiles of the urine, which could thus serve as an honest signal of social status.

While conducting NMR-based metabolomic analyses of FHM urine to evaluate differential responses to chemical stressors, we observed a large variation in the urine abundance, and composition of urinary metabolites in the males [24]. We also observed that the stored urine volume co-varied with the expression of androgen-dependent secondary sexual characteristics, which are good indicators of sexual maturity and social status. Based on these preliminary observations, we developed and tested the following hypotheses: 1) expression of secondary sexual characteristics (SSCs), sperm abundance and stored urine quantity are responsive to territorial status, 2) patterns of urine release are dependent on the social context (male-male vs. male-female interaction), and 3) metabolite composition of the urine is linked, and responsive to territorial status.

## Materials and Methods

### 2.1. Experimental animals

Fathead minnows were bred and maintained at the U.S. Environmental Protection Agency (Duluth, Minnesota, USA). Rearing and experimental conditions followed those outlined by [25]. We modified the original protocol so that males and females, which are usually reared in mixed-sex groups (ca. 40 fish per 40 L aquarium), had no access to artificial substrates (nesting tiles) prior to onset of the experiments. All fish used in this experiment were sexually mature (6–7 months old). Sexually mature males were identified based on the presence of male-specific SSCs – nuptial tubercles above the mouth and a dorsal pad (mucus producing tissue on the head). Mature females were identified based on the presence of ovipositor and distended abdomen. Phenotypic sex was verified based on gonad morphology at the end of each experiment. All activities involving animals were conducted in accordance with the federal regulations and animal welfare guidelines as outlined in the protocols approved by the U.S. Environmental Protection Agency's and University of St. Thomas' Institutional Animal Care and Use Committees (IACUCs). The protocols specific to this study were approved by U.S. Environmental Protection Agency's and University of St. Thomas' (Permit number 51) Institutional Animal Care and Use Committees. All animal manipulations (injections) were performed under tricaine methanesulfonate (MS-222) anesthesia, and all efforts were made to minimize stress.

### 2.2. Experiment 1: Effects of sex and territorial status on urine storage, secondary sex characteristics, and sperm production

Two d prior to the start of the experiment one male was placed in each of 45 5- L holding aquaria and held in isolation. The same procedure was followed for females. During this time fish were fed frozen brine shrimp (Sally's, San Francisco Bay Brand Inc., Newark, CA, USA) *ad libitum*. After 48 h of isolation, on Day 1 of the experiment we measured stored urine volume in females. We also measured males' stored urine and sperm volumes, mass and total length. To measure urine and sperm volumes, the animals' abdomen was gently pressed and sperm or urine collected with borosilicate glass capillary tubes. Volumes were calculated based on the height of the sperm and/or urine column within capillary tubes and the internal diameter of the tube. On Day 1, we also scored prominence of secondary SSCs in males using the method of Danylchuk and Tonn [15]. Briefly, development of nuptial tubercles and dorsal pad (male-specific SSCs expressed only in sexually mature males) were scored using a well-established qualitative scale that ranged from 0 (no pad; no tubercles) to 3 (sharp, prominent tubercles; dorsal pad wide and thick forming a sharp nape behind head). We summed tubercle and dorsal pad scores to obtain a total SSC score (maximum possible score  = 6).

After these measurements were completed, we combined animals in two scenarios. In the first (*‘non-competitive scenario’*), a single male was placed in an experimental tank (10 L) with two sexually mature females and a nest (an 8-cm section of a cross-sectioned polyvinyl chloride plastic pipe [diameter  = 10 cm]). In the second (*‘competitive scenario’*), two males were placed into an experimental tank containing two mature females and a nest. We tested 15 replicates of each scenario. The tips of the caudal fins of all males in both scenarios were clipped prior to the placement in the experimental aquaria (bottom or top clips were assigned randomly) to distinguish individuals.

Identification of T vs. NT males commenced after 24 h had elapsed from the time the fish were introduced into experimental aquaria. To accomplish this, each fish was observed for one 5-minute period each day for 7 days; all behavioral observations were performed between 10.00 h and 14.00 h. Fish that were observed to spend the majority of their time in a nest, and exhibited either nest defense behavior or nest-tending behaviors, were categorized as a ‘territorial’ (for behavioral protocol details see [25]). Conversely, fish that did not display such behaviors were categorized as non-territorial. Note that for the purposes of this report, territorial behavior (T) is considered synonymous with dominant behavior, and non-territorial (NT) behavior is considered synonymous with subordinate behavior. After 7 d, fish were euthanized with an overdose of MS-222 (0.1 g MS-222/L). Mass, length, stored urine, and sperm volumes were measured.

Statistical analyses were performed using STATISTICA 10 (StatSoft Inc., Tulsa, OK, USA). Association (contingency) between sex and presence of stored urine was tested using Fisher's Exact Test. Effects of time (before vs. after social interaction) and territorial status on body mass, urine and sperm volumes were assessed using Repeated Measures ANOVA. Effects of territorial status on change in SSC scores (calculated by deducting Day 7 score from Day 1 score) were assessed using Kruskal-Wallis ANOVA. Differences in this and the other experiments were considered significant at p<0.05 unless noted differently.

### 2.3. Experiment 2: Effects of sex, territorial status and social context on urine release patterns

Fathead minnows were exposed to different social stimuli and the frequency of urine release was recorded. The test subjects included: 1) sexually mature T males (n = 8), 2) sexually mature NT males (n = 8), and 3) sexually mature females (n = 8). To visualize urine pulses the test subjects were first briefly anesthetized with MS-222, and injected 20 uL of patent blue violet (100 mg/ml in 0.9% NaCl, Sigma-Aldrich Inc., USA), following procedures described by Appelt and Sorensen [5]. This dye is commonly used for human medical procedures as a coloring agent and does not have adverse effects on health of fish [5]. Before the onset of behavioral trials fish were allowed to recover from injections and anesthesia for 45 minutes, during which time they were observed to ensure that they were able to function in the same manner as observed prior to anesthesia and injection.

Once recovered, the color-injected males were tested within one of the following social settings: 1) alone, 2) paired with a sexually-mature female, and 3) paired with a sexually-mature male of opposite social status (T or NT). Female fish were tested with one of the following social settings: 1) alone, 2) paired with a sexually-mature female, 3) paired with a sexually-mature T male, and 4) paired with a sexually-mature NT male. To identify T vs. NT males for the behavioral trials, males were held in competitive scenarios (see Experiment 1 for description) for 24 h prior to the onset of experiments. Territorial status of individuals was assigned using the criteria described in Experiment 1.

Test subjects were placed in the 5 L aquaria (each containing one nest tile) with the stimulus fish for 20 minutes, and frequencies of urine pulses and aggressive behaviors were recorded. The order of social setting testing was randomized, and each fish was tested in a maximum of three (males) or four (females) different settings. Overall, we obtained data for eight trials for each of the treatment combinations. The statistical significance of effects of the social stimuli on the urine release was evaluated separately for males and females using Friedman's ANOVA. Post- hoc comparisons of interest were performed using Wilcoxon's Matched Pairs Test.

### 2.4. Experiment 3: Effects of territorial status on the composition of male urinary metabolites

The goal of Experiment 3 was to determine whether there were differences in the urinary metabolite profiles between the T and NT males before (Day 0) and after (Day 8) territory acquisition, and thus establishment of the social hierarchies. In addition, we identified several of the specific metabolites accounting for observed differences in the metabolomic profiles of T versus NT individuals.

#### 2.4.1. Sample generation

The design of the behavioral experiments was identical to that of Experiment 1, with two exceptions: 1) experiment was 8 days long, and 2) we did not include the non-competitive scenario in this assessment. We collected urine from 24 fish (a total of 12 replicate trials) on Day 0 (before territories were established) and Day 8 (after the territories were established). Mass, total length, stored urine volume, sperm volume and SSCs were evaluated on these days. In order to compare metabolite composition across time (i.e., Day 0 versus Day 8) for a given group of individuals, we omitted samples from fish that did not yield urine on both sampling days. Also, we omitted both Day 0 and Day 8 samples from one fish, which was identified as a strong outlier based on a preliminary model (the criteria used for exclusion are detailed below). This left n = 8 for four classes of samples: T (0), T (8), NT (0), and NT (8). T (0) and T (8) were urine samples from territorial males taken on Day 0 (before they became territorial) and Day 8 (after they became territorial), respectively. As such, this experiment had a temporal component, because, for each of these eight dominant males, a urine sample was collected both before (T (0)) and after (T (8)) territory/social status was established. A similar designation was used for urine samples from the eight subordinate/non-territorial males (NT (0) and NT (8)).

#### 2.4.2. Sample collection

On Day 0 (before territory establishment) after anesthesia with MS-222, urine was expelled from male fish by applying gentle pressure on the abdomen, collected directly from the cloaca into non-heparinized microcapillary tubes, and snap frozen at −80°C. Once the urine sampling was completed, fish were returned to their source tanks containing clean, aerated water and allowed to recover from the anesthesia and handling for 2 h before placement in the experimental tanks. Once recovered, two males were placed into an experimental tank containing two mature females and a nest. On day 8, urine samples were collected again from the same individuals. All urine samples were collected using multiple capillary tubes in 5 µL increments to avoid pollution with the sperm and seminal fluid. Only the first 5–10 µl of a sample was used for metabolomics. We have tested the suitability of this sampling strategy in our past experiments, and were able to show that this is an adequate approach to avoiding sperm contamination (data not shown).

#### 2.4.3. Preparation of samples for metabolomic analysis

Urine samples from individual fish were lyophilized for 8 h to remove water. Subsequently, each sample was reconstituted in 90 μL of sodium phosphate-buffered deuterium oxide (0.1 M, pH 7.4) containing 25 μM 3-(trimethylsilyl) propionate-2,2,3,3-d_4_ (TSP). Samples were then vortexed briefly, and centrifuged at 10,600× g for 10 min at 4°C to remove any insoluble components. The resulting supernatants were pipetted into 3 mm Shigemi microtubes (Shigemi, Inc., Allison Park, PA, USA) and analyzed by ^1^H-NMR spectroscopy.

#### 2.4.4. NMR spectroscopy

One dimensional ^1^H-NMR spectra were acquired on a 600 MHz Agilent Inova (599.76 MHz, ^1^H) spectrometer equipped with a cryogenic triple-resonance probe. All spectra were collected at 25°C using a standard presaturation pulse sequence For each spectrum, 2048 transients were collected into 14 k points using a spectral width of 7200 Hz and an acquisition time of 2 s. Suppression of the residual water resonance was achieved by a 1-s pre-saturation delay at field strength of 40 Hz. Metabolite assignments were made based on previously reported chemical shift values and multiplicities [24].

#### 2.4.5. Processing and multivariate analysis of NMR data

Spectra were processed using ACD/1D NMR Manager (Advanced Chemistry Development, Toronto, Canada). Specifically, spectra were zero-filled to 32,768 points, and exponential line broadening of 1.0 Hz was applied prior to Fourier transformation. An automated routine was used to phase and baseline-correct each spectrum, which was then referenced to TSP (at 0.0 ppm). Next, the spectra (0.50–10.00 ppm) were segmented into 0.005 ppm-wide bins and imported into Microsoft® Excel (Microsoft Corporation, Redmond, WA). Within Excel the region 3.29–3.40 ppm was omitted to remove the resonance from an unexplained contamination of methanol, and the region 4.20–5.50 ppm was omitted to remove the residual water resonance. The remaining bins were then normalized within Excel to achieve unit total intensity for each spectrum. This Excel spreadsheet of binned spectra was then imported into SIMCA-P+ (Umetrics Inc., Umea, Sweden) for multivariate data analysis (which was conducted on mean-centered and Pareto scaled bins.) First, principal components analysis (PCA) was used to screen for outliers in the dataset. As mentioned above, the urine samples from one fish (both day 0 and day 8 samples) were found to be outliers using the Hotelling's T^2^ test at the 95% confidence interval for a scores plot of the first two components). Thus, these two samples were removed prior to further analysis. Next, the dataset was submitted to partial least squares discriminant analysis (PLS DA) for assessing the impact of territorial status on urinary metabolite profiles at both sampling days. The permutation testing routine (Wold reference) within SIMCA-P+ was used to confirm the validity of the PLS-DA model.

The Excel spreadsheet of binned, edited, and normalized spectra was also used to construct “t-test filtered difference spectra,” which aided in identification of specific metabolites that were affected by social status and sampling day. In addition, these difference spectra were used to further compare the relative impacts of social status on the urinary metabolite profiles. In order to generate these difference spectra, an “average class spectrum” was first calculated by averaging the binned spectra across all class members (note that class was defined by social status and sampling day). Next, the difference spectrum for each comparison (e.g., T (0) vs. NT (0)) was generated by subtracting the averaged bins of one class (e.g., NT (0) from those of another class (e.g., T (0)). Then, a t-test was conducted on each bin to determine if the average for the one class differed significantly from that of the other class, using a p-value <0.05. If not, the bin value for the difference spectrum was replaced with a zero. To greatly reduce the rate of false positives, any single isolated bin that passed the t-test (without an adjacent bin also passing) was replaced with a zero (i.e., it was rejected), because legitimate metabolite peaks span more than one bin at this bin size. To further reduce false positives, any occurrences of two (and only two) adjacent bins with opposite arithmetic sign were replaced with zeros, because this outcome is incompatible with NMR peak shapes. For more details on generating these difference spectra, and on limiting their false positives rates, see [26].

It is important to note that the t-test filtered difference spectra were used to help identify which metabolites might be social status- and time-dependent (i.e., Day 0 or Day 8). After identification in this manner, social status and time dependency was either confirmed or rejected by integrating the area of a given metabolite's most abundant and/or most isolated peak for each class member. These integrated peak areas were calculated in Excel using the same spreadsheet of binned, edited, and normalized spectra described above. Then, a univariant t-test was conducted to assess social status and time dependency using a p value <0.05. All specific peaks and metabolites discussed herein as changing due to social status or sampling day passed this univariate test.

## Results

### 3.1. Experiment 1: Effects of sex and territorial status on urine storage, SSCs and sperm production

There were no significant differences in the initial lengths of territorial males tested in a non-competitive scenario (TNC) (67.13±5.44 mm, mean ± SEM), territorial males tested in a competitive scenario (T) (69.07±6.3 mm), and non-territorial males tested in a competitive scenario (NT) (63.85±5 mm). Similarly, there were no significant differences in the initial total masses of the TNC (4.22±1.14 g), T (4.62±1.33 g), and NT (3.61±0.85 g) males. All three groups experienced some body mass loss over the 7-d experimental period, and there was a significant interaction between time and social status/scenario treatments on loss of mass. The TNC males experienced the least mass loss (7.6%), and the T and NT males tested in a competitive scenario experienced higher body mass losses (13% and 12%, respectively).

**Figure 1:**
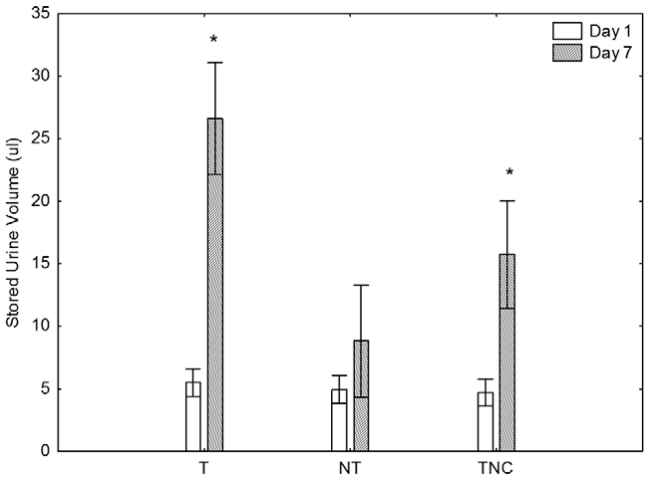
Effects of territorial status on stored urine volumes. Urine volumes (mean ± SEM) measured in territorial (T) and non-territorial (NT) males under a competitive scenario, and in territorial males (TNC) in a non-competitive scenario. Measurements were performed before (Day 1), and after (Day 7) territory establishment. Repeated measures ANOVA indicated that urine volumes were significantly affected by territorial status, time of sampling and that the two factors interacted (* p<0.05).

There was a strong, statistically significant association between sex and presence of stored urine (Fisher's Exact Test, p<0.0001). Expressable urine was present in females only once out of 15 trials (ca 7%), whereas in males it was present in 43 of 45 (ca 96%) sampling events. Stored urine volume on Day 1 was comparable in the three male groups (T, NT and TNC), but by the day 7 there were significant increases in the stored urine volumes of T males tested in competitive (ca 5-fold) or noncompetitive (ca 3-fold) scenarios (Figure 1). In contrast, the NT males did not show a significant increase in the stored urine volume.

**Figure 2:**
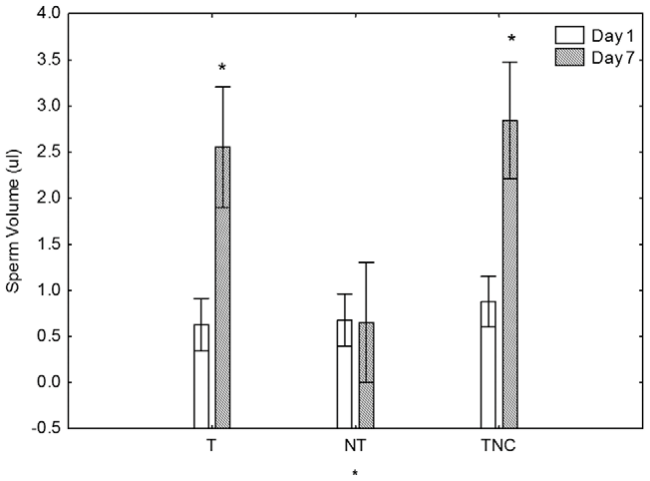
Effects of territorial status on stored sperm volumes. Sperm volumes (mean ± SEM) measured in territorial (T) and non-territorial (NT) males under competitive scenario, and in territorial males (TNC) in a non-competitive scenario. Measurements were performed before (Day 1), and after (Day 7) territory establishment. Repeated measures ANOVA indicated that sperm volumes were significantly affected by territorial status, time of sampling and that the two factors interacted (* p<0.1).

**Figure 3:**
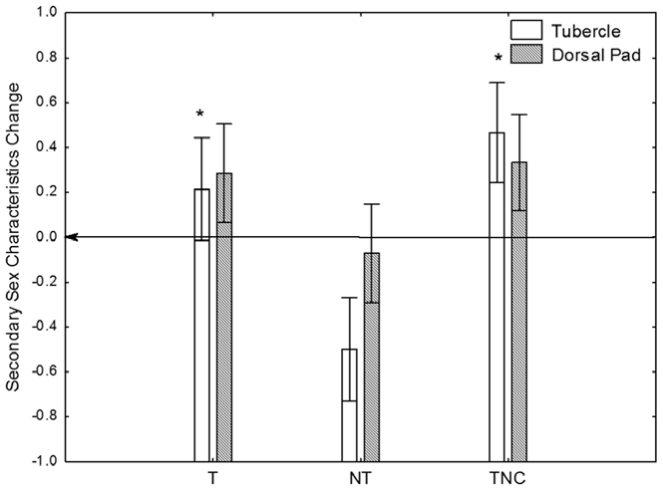
Effects of territorial status on male-specific secondary sex characteristics. Change in the secondary sexual characteristics score (Change =  Day 7 score – Day 1 score) before and after territory and social hierarchy establishment. T- Territorial males tested in a competitive scenario, NT- not-territorial males tested in a competitive scenario, and TNC- territorial males tested in a non-competitive scenario. Kruskal-Wallis ANOVA indicated that there was a significant effect of territorial status on the tubercle score change (* p<0.05), but not on the dorsal pad score.

The sperm volume data showed the same trend as urine data, with T and TNC males experiencing ca 5-fold increases in the sperm volume, and NT males exhibiting no change (Figure 2). We also observed statistically significant increases in the tubercle scores in T and TNC males, but not in NT males, again following the patterns observed for urine and sperm (Figure 3). While the changes in dorsal pad scores were not statistically significant, they followed the same general pattern as the tubercles. When the data for all three treatments (T, NT and TNC) were pooled we observed that urine volume correlated positively (Spearman Rank Order Test, p<0.05) with sperm, and tubercle and dorsal pad scores on both sampling days.

### 3.2. Experiment 2: Effects of sex, territorial status and social context on urine release patterns

**Figure 4:**
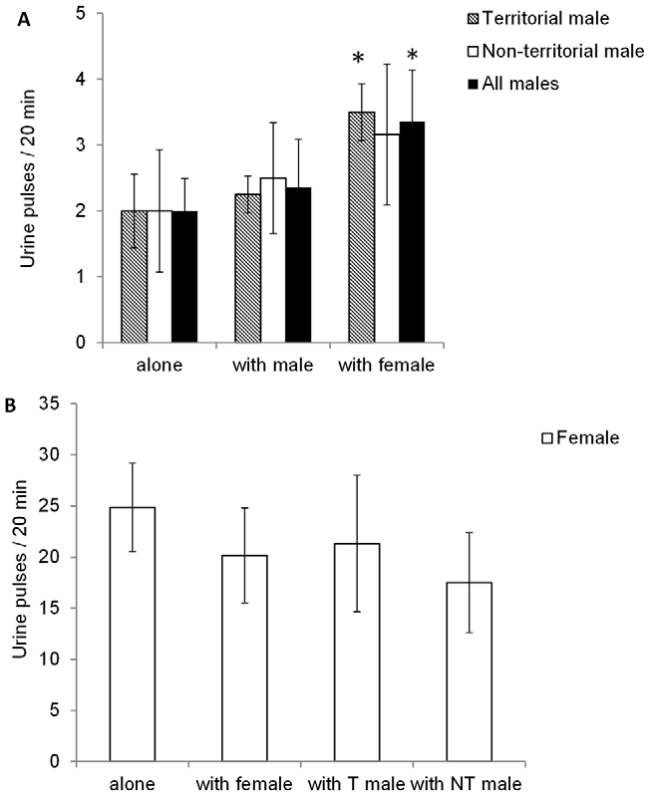
Effects of sex, territorial status and social surroundings on urine release patterns. Urination frequency of A) territorial (T) and non-territorial (NT) males and B) females tested in different social situations. Males were observed while alone (n = 8), paired with female (n = 8), or paired with a male of opposite territorial status (n = 8). Females were obserwhed while alone, paired with a female or a male (T and NT male data pooled together) (n = 6–8 per treatment), * p<0.05.

There were no significant differences between the frequency of urine release by T and NT males when tested alone or in the presence of other males (Figure 4A). In contrast, the males released urine with higher frequency in the presence of females. While both T and NT males increased urination rates in presence of females, post-hoc testing indicated that these differences were significant only for the T males. Females did not show any changes in urination frequency when tested alone and/or with other females or males (Figure 4B). It is noteworthy that male and female urination frequencies were significantly different, with females urinating approximately 10 times more frequently than males. The frequency of urine release and frequency of performance of aggressive behaviors by males were negatively correlated (Spearman Rank Correlation, Spearman r = −0.6238, 95% confidence interval: −0.8786 to −0.09272, p = 0.0227).

### 3.3. Experiment 3: Effects of territorial status on the composition of male urinary metabolites

The results of Experiment 3 regarding stored urine volume and SSCs replicated the findings of Experiment 1. Briefly, in Experiment 3, the stored urine volume and SSCs prior to territory establishment were comparable in T and NT males, but after the territory establishment there were prominent increases in the stored urine volumes and SSCs of T, but not NT males. Non-territorial males exhibited a 55.6% (−7.63, 1137.5) increase, and T males a 381% (121, 590) increase in stored urine volume; median (1^st^quartile, 3^rd^ quartile). Similarly, NT males exhibited a 0% (0, 58) increase, and T males a 33% (10, 100) increase in the combined SSC score; median (1^st^quartile, 3^rd^ quartile)).

**Figure 5:**
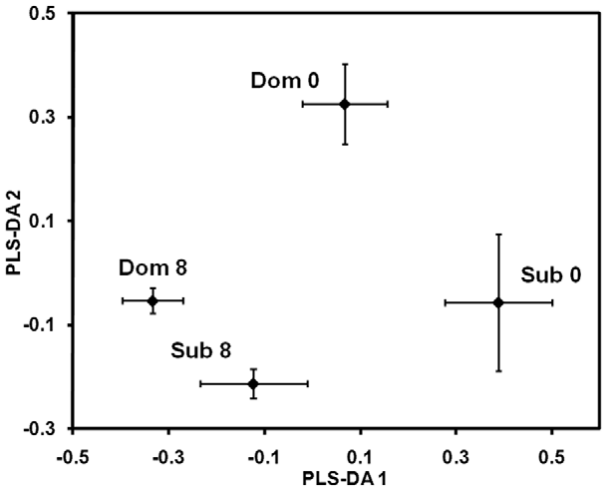
Urinary metabolite profiles reflect individual's potential to acquire territory, and its present territorial status. Two-component scores plot from partial least squares discriminant analysis (PLS-DA) of binned NMR spectra of urine from the territorial (Dom) and non-territorial (Sub) male fathead minnows collected before (0) and after (8) territory establishment. Each marker is the mean score value, shown with its associated standard error.

A two-component scores plot from a PLS-DA model built for the metabolite profiles, as characterized by NMR spectra, of four classes of fish is shown in Figure 5. This plot reveals a significant degree of separation of classes according to both territorial status, and the time at which the animals were sampled (before vs. after territory establishment). It is interesting to note a greater similarity in metabolite profiles of territorial and non-territorial males after territoriality was established, as compared to beforehand (i.e., the distance between the T (8) and NT (8) markers is less than for the T (0) and NT (0) markers).

**Figure 6:**
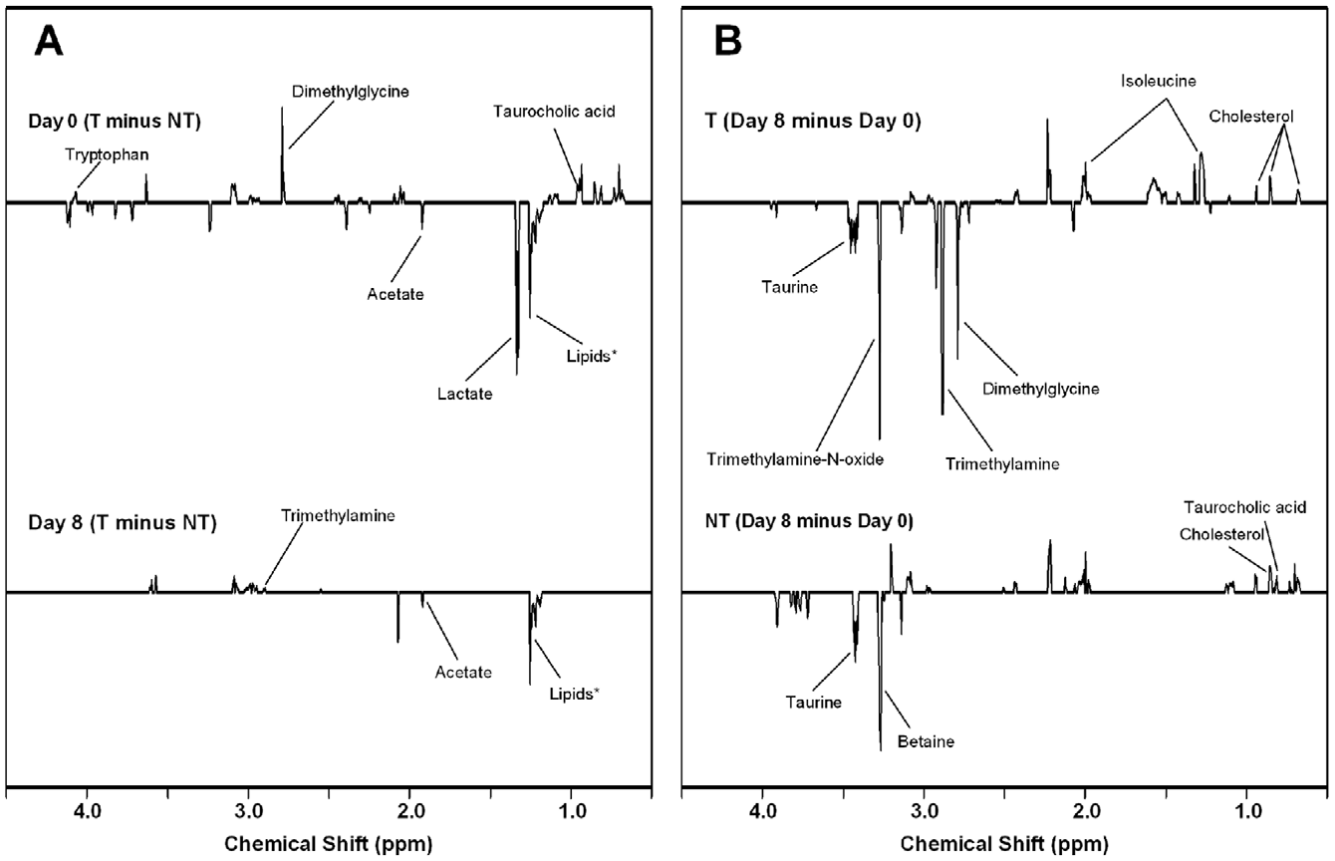
Identification of specific metabolites associated with the social status and the acquisition of the territories. Average NMR difference spectra (t-test filtered) from male urine, all of which are displayed using the same Y-axis scale. A) Top is non-territorial (NT) day 0 subtracted from territorial (T) day 0; bottom is NT day 8 subtracted from T day 8. Thus, positive-going peaks correspond to metabolites that are greater in the territorial male's spectra, while negative going peaks correspond to metabolites that are greater in the non-territorial male's spectra. B) Top is T day 0 subtracted from T day 8; bottom is NT day 0 subtracted from NT day 8. Thus, positive-going peaks correspond to metabolites that are greater in the day 8 spectra, while negative going peaks correspond to metabolites that are greater in the day 0 spectra.

The t-test filtered difference spectra (Figure 6) allow further characterization of metabolite profiles of the T and NT males at the two sampling times, including identification of which specific metabolites were changed, and to what extent. While we could not reliably and confidently identify all metabolites that were different, we did identify 19 metabolites, and the chemical shift peaks for those are named and labeled in Fig. 6. Figure 6A indicates that there are substantial differences in the abundance of metabolites between T and NT males, and that the differences are detectable before (Day 0), as well as after the territory acquisition (Day 8). Nevertheless, it is important to note that differences in metabolite profiles between T and NT males on Day 0 vs. Day 8 are not driven by an identical combination of metabolites (i.e., a different set of peaks are observed in the two difference spectra in Figure 6A). In addition, it is noteworthy that there are more peaks, and more-intense peaks, observed in the difference spectrum for Day 0 (top trace in Figure 6A) than for Day 8 (bottom trace in Figure 6A). This indicates a greater difference in metabolite profiles of territorial and non-territorial males before territoriality was established (as compared to afterward). Note that this is consistent with observations from the PLS-DA scores plot in Figure 5.

In Figure 6B, t-test filtered difference spectra were constructed in a different fashion. Specifically, in the top trace of Figure 6B, the average spectrum for territorial males collected at Day 0 was subtracted from that of the territorial males at Day 8. The bottom trace is the same for the non-territorial males. These two traces provide an indication of the extent of change in metabolite profiles for a given group of fish over time (i.e., before and after territorial acquisition). Interestingly, while the two groups of fish experience some of the same metabolite changes, the territorial males (top trace) experience a somewhat greater overall change over time.

## Discussion

The present study shows that the acquisition and maintenance of territory was associated with increased stored urine and sperm volumes, and augmented prominence of androgen-dependent SSCs in sexually mature FHM males. The increased sperm investment in territorial FHM males, at first consideration, seems to be contrary to the hypothesis that there should be a trade-off between mating role and ejaculate investments, with subordinate males investing more into ejaculation quality than dominant males [27]. In several species [28], [29], including cichlids [30], there is a positive relationship between a dominant status and ejaculate quantity and quality. Such relationships are typically observed in species where subordinate males realize fitness benefits, not through sperm competition, but through inclusive fitness [31] or by adopting a ‘wait to mate’ strategy, investing in survival until the dominant male is displaced or dies [32]. Fathead minnows practice a ‘wait to mate’ strategy [15], and thus the sperm volume dynamics we observed are consistent with expectations.

The finding that expression of SSCs (tubercles and dorsal pads) was responsive to territorial status supports the proposition that androgen-dependent morphological traits are regulated according to individual's resource holding potential [33]. In a social system in which male-male agonistic interactions are frequent, males that display characters that do not correspond to their resource holding potential may incur high costs by provoking agonistic confrontations that they may not be able to win. Thus, androgen-dependent ornaments and behaviors are expected to be honest signals of male quality. In the case of FHMs, SSCs appear to signal two aspects of male quality – sexual maturity, and territorial status. The most obvious proximate mechanism responsible for enhancement of male SSCs is activation of the androgen receptor. In FHM, secondary sex characteristics have been shown to correlate with sexual maturation and increased levels of endogenous androgens [34]. Several manipulative studies have shown that synthetic androgens can enhance the expression of SSCs in FHMs [25], [35], and increase agonistic behaviors and the ability of males to acquire a territory [25]. Whether SSCs are enhanced directly by territorial status, or indirectly by increased interactions with females because of territoriality has yet to be determined.

Given similar patterns of response to territorial acquisition in stored urine volume, SSC expression and expressable sperm volume, it is tempting to hypothesize that the proximate and ultimate factors that drive these changes in territorial males may be the same. While this assumption is not foolproof – we know, for example, that observed sperm volume increases may be a result of more intense interactions of territorial males with females, and may be primarily stimulated by pheromonal progestins [36] – it is conceivable that the increased urine storage is stimulated by the increase in circulating androgens associated with the social challenge during the acquisition of territories. There is evidence that changes in androgen levels increase bladder muscular mass, and affect the urination patterns of other vertebrates such as rodents [37]. Thus, it is plausible that androgens might promote urine storage in fish. There is, in fact, some evidence of involvement of androgens in urine formation and osmoregulation in marine fish [38]. Additional support for the idea that urine storage may be androgen-dependent comes from our observations that urine storage was a male-specific phenomenon, and that females managed urine differently. While males stored urine, this was much less apparent in females, who also urinated with a much higher frequency. Similar patterns have also been noted in tilapia [39]. Furthermore, we have observed that sexually immature males exhibit female-like patterns of urine storage (unpublished data, Martinovic-Weigelt), again suggesting that androgens may be involved.

Data from the present study show that territorial males not only have higher stored volumes of urine, but that they preferentially increase the frequency of urination in the presence of sexually mature females. Based on this, we propose that urine-mediated chemo-signalling might be used to advertise males' territorial status to females. The added support for urination being a female-oriented behavior, and not male-oriented agonism, comes from the observation that males' urine release frequency and aggressive behaviors towards other males were inversly correlated. Nevertheless, one could hypothesize that aggressive behaviors and urine release do not correlate because urine is released prior to conflict, to prevent escalation of the conflict. If this were the case, one would expect that urinations would occur with higher frequency in presence of other males, who are most commonly engaged in the agonistic encounters, but we did not observe this.

Holding urine and timing its release based on the female presence/reproductive status is a subject to physiological constraints because one of the osmoregulatory strategies deployed by the freshwater fish is to produce copious and dilute urine. Hence, it is likely that the ability to retain urine and time its release based on social setting, ultimately is constrained by osmoregulatory needs. In fact, metabolite changes (e.g., trimethylamine, trimethylamine-N-oxide, dimethylglycine) indicate that osmoregulation and nitrogenous waste formation differed some between T and NT males. Unger [16] noted that territorial male FHMs nesting for long periods retain water to conceal dry weight loss. He hypothesized that this is a strategy by which T males who held territories for a long time (and have relatively low resource holding potential due to the “exhaustion” associated with nest defense and repeated spawning and egg care), mimic more vigorous newly arrived T males, and thereby lessen the probability of being displaced from their nests. Consistent with this, we found that T males experienced a substantial weight loss during the test period. This fits with Unger's idea that size might need to be maintained by water retention, in order to compensate for dry weight loss. This suggests that there may be multiple forces selecting for urine retention in males. Paradoxically, urine retention might serve as both an honest signal of territoriality (*via* chemical signalling), and as a dishonest signal *via* size maintenance in spite of dry weight loss.

Organisms that use chemical signalling to convey information about their state usually achieve this by varying qualitative composition and/or concentrations of chemicals in the chemo-signalling medium [7], [8], [13]. We found that T and NT males had distinct urinary metabolomic profiles. Our data also show that the urinary metabolome of solitary individuals changed after pairing with males and females and competition for territories. This is to be expected, given new physiological and behavioral requirements imposed on the individuals, as well as possible pheromonal influence of conspecifics. An unforeseen finding was that one could discern future territorial status of males, based on their initial metabolomic profiles (i.e., Day 0). These results indicate dominant males may be intrinsically different, and indicate that the roles that males adopt (dominant or subordinate) may be at least partly influenced by genetic factors [40], [41]. Overli et al. [42] have demonstrated that distinct behavioral–physiological stress coping styles are present in teleost fish, and these coping characteristics influence both social rank and levels of aggression. Whether the differences in initial urinary profiles of male FHMs were driven by their past experiences (e.g., social experience, nutritional status), or have a genetic basis and reflect two distinct coping styles remains unknown; additional experimentation is required to examine these alternative scenarios.

In order to advance our understanding of the potential of urinary chemo-signalling in FHM, and to start elucidating which chemicals might be involved, we identified a number of metabolites that were differentially expressed between T and NT individuals. One of the chemicals that may be of special interest is trimethylamine; it was one of the few chemicals that was significantly different between T and NT males after the territory acquisition, and its concentrations changed dramatically in T, but not NT fish over the duration of the experiment. Trimethylamine is a volatile amine, has a strong “fishy” odor in low concentrations and an ammonia-like odor at higher concentrations. It is an agonist for trace amine-associated receptors (TAARs) [43]. In mice, TAARs are selectively expressed in the olfactory epithelium, and thought to facilitate detection of social cues [44]. For example, TAARs in mice can recognize volatile amines found in urine, one linked to stress and two linked to chemicals enriched in females vs. males (trimethylamine is one of them) [43]. In fish, TAARs are expressed in subsets of olfactory receptor neurons indicating their role in olfaction [45]. Furthermore, phylogenetic analyses have shown that many lineage-specific gene gains and losses occurred in the teleost fish TAARs suggesting that TAARs are used for detecting species-specific chemicals such as pheromones [46]. Our finding that metabolic profiles of T and NT males differ in regard to volatile amine qualitative and quantitative composition, suggest that volatile amines and TAARs may play a role in signalling of the territorial status in fish.

Given our finding that one could “predict” future territorial status of males, based on their initial metabolomic profiles, it is also important to examine the potential role of metabolites that were different prior to territory acquisition and social interaction (Day 0) as putative signals of dominance. One chemical that was more abundant in dominant individuals before the territory acquisition was a bile acid – taurocholic acid. Bile acids are known to function as odorants in fish. Several species have been shown to be highly sensitive to bile acids, responding to concentrations less than 10^−9^ mol/L, which would be consistent with a role for bile acids as olfactory signals [47]–[48]. Because of this, we quantified taurocholic acid and found that urine contains micromolar concentrations, indicating that it could serve as a chemical signal, at least in the close proximity of the males. Interestingly, the focused quantification also suggested that this bile acid was also more abundant in dominant males on Day 8 (but not statistically significant). Many have suggested that bile acids may play a signalling role in both intra- and interspecies interactions [49], [50], but the only fish species in which the role of the bile acids in chemical signalling has been conclusively elucidated is lamprey. In this species bile acids act as pheromones involved in migratory and reproductive behaviors [50], [51]. Taurocholic acid is an interesting candidate chemical signal of territoriality for at least a couple more reasons. First, taurocholic acid is a conjugate of cholic acid with taurine. Taurine augments the effects of sex steroids in the promotion of spermatogonial proliferation and/or meiosis and plays important roles in spermatogenesis in eel [52]. Second, in mammals taurine is known to promote social interactions and reduce 5-hydroxytryptamine [53]; 5-hydroxytryptamine modulates aggressive behaviors in many species including fish [54]. There is some indication that stress resulting from subordination due to low social status promotes bile retention (and thus bile acid retention) in subordinate cichlid fish (*Archocentrus nigrofasciatum*) [55]. The release of the taurocholic acid in our study did not follow this pattern; it was increased in NT males after they have gone through such social interactions.

Tryptophan was also differentially expressed on day 0 in the present study, and is of interest because it too has been linked to aggressive behavior and territoriality [56]. The precise role of tryptophan and its metabolites in fish chemical communication has not been investigated, although it might be involved in feeding and prey recognition [57]. Given the potential of tryptophan (and/or its metabolite, 5-hydroxytrytophan) to serve as an honest signal of aggression, further investigation as to its role(s) relative to chemical signalling in fish is warranted. More specific identification of lipids is planned and may provide additional insight in identities of putative chemo-signals of territoriality. Further work should be conducted with the groups of above candidate chemicals to elaborate whether females are receiving and actively using these putative signals.

In conclusion, we demonstrate that expression of SSCs, sperm abundance and urine quantity are responsive to territorial status in FHM males. The finding that the patterns of urine release differs between T and NT males, and are associated with female presence suggests that females are the main target of the putative urinary signals. Finally, metabolite composition of the urine was linked to, and responsive to both current and future territorial status. Bile acids and volatile amines emerge as potential chemical signals of social status.
